# Comparing the effectiveness of master’s-level clinicians and peer supports for the delivery of attachment and biobehavioral catchup

**DOI:** 10.1017/cts.2026.10764

**Published:** 2026-06-05

**Authors:** Elisabeth Conradt, Claire Dahl, Kristen Cotton, Erin Hoffman, Evette Horton, Mary Dozier, Danielle S. Roubinov

**Affiliations:** 1 Department of Psychiatry, School of Medicine, Duke University, Durham, NC, USA; 2 University of Delaware, Newark, DE, USA; 3 The University of North Carolina, Chapel Hill, NC, USA; 4 Psychological and Brain Sciences, University of Delaware, Chapel Hill, NC, USA; 5 Department of Psychiatry, University of North Carolina Chapel Hill, NC, USA

**Keywords:** Comparative effectiveness, peer support, foster care, parental sensitivity

## Abstract

The goal of this study was a re-analysis of a randomized controlled trial evaluating the effectiveness of Attachment and Biobehavioral Catchup (ABC), an early childhood parenting intervention. We compared the effectiveness of ABC for enhancing parental sensitivity and child behavior among 79 dyads who received the intervention as delivered by peer support specialists (n = 49 dyads) or master’s-level clinicians (n = 30 dyads). Peer support parent coaches were non-inferior at enhancing parental sensitivity and parental intrusiveness as parent coaches with a master’s degree. This research has important clinical implications for increasing access to ABC for parents involved in the child welfare system.

Approximately 75% of adults in treatment for substance use disorders are parents, and almost 19 million children in the United States live with a parent with a substance use disorder (SUD) [1]. Parents with a SUD are frequently involved in the child welfare system and are mandated to receive parenting support to maintain custody of their child [2]. However, the quality and efficacy of these parenting treatments vary substantially; this is a critical issue for three main reasons. First, children with substance exposure tend to have more challenges with behavior regulation than children without exposure [3–6]. Second, parents involved in the child welfare system report that stigmatizing experiences make it challenging for them to engage in parenting treatments, and parents have reported that they would prefer to receive parenting support from a peer rather than a mental health clinician with an advanced degree [7]. Third, behavioral health workforce shortage issues contribute to an insufficient number of trained providers to meet the demand within SUD recovery programs [8]. Thus, there is a critical gap in research-backed interventions that are rigorous, high-quality, destigmatizing, and two-generation to improve parenting behavior.

Attachment and Biobehavioral Catchup (ABC) is an evidence-based 10-session home visiting intervention for children who have experienced early adversity [9]. Systematic reviews have shown that ABC significantly improves parenting sensitivity and children’s self-regulation and behavioral outcomes [9,10]. ABC was designed for parents involved in the child welfare system, many of whom are also in treatment for substance use disorders, because ABC is concrete, behavioral, and supports early childhood behavioral regulation. While ABC is typically delivered by master’s-level clinicians, parent coaches are not required to be licensed to conduct ABC, and years of advanced training does not predict fidelity to the ABC model [9–11].

All individuals receive the same rigorous training to become a certified ABC parent coach. Specialized clinical credentials are not required, and ABC training and certification processes are not altered for those with different levels of experience. In fact, many ABC parent coaches do not have specialized clinical training and are instead considered parent peer supports, including parents who have previously received ABC and “graduated” from the program. However, the comparative effectiveness of the “parents-as-peers” ABC parent coaches compared to the master’s-level clinician coaches has never been examined. By addressing this question, we aim to help understand how to best address the needs of parents in SUD treatment programs and their offspring: Should limited resources be used to train master’s-level clinicians? Or should resources be used to train peer specialist parent coaches who are more widely available and who may also be highly trusted by patients but who lack traditional clinical credentials? The aim of this study was to examine non-inferiority of peer specialists compared to master’s-level clinicians for maternal sensitivity, intrusiveness, and positive regard, hypothesized mechanisms of effect in the ABC model [9–11]. We also evaluated whether non-masters-level provider types are non-inferior to master’s-level provider types on attachment organization and child inhibitory control.

## Methods

### Participants

The analytic sample included 79 parent–child dyads recruited for a randomized controlled trial of ABC in a large city in the Mid-Atlantic region. Participants were referred to the study by Child Protective Services (CPS) as part of a diversion from foster care program for families that had been identified by or referred to CPS for risk of neglect or abuse. A total of 101 parent–child dyads completed at least one ABC session with a parent coach (6 peer coaches and 6 master’s-level coaches). Of the 101 parent–child dyads who completed an ABC session, only 79 dyads completed a post-intervention session (*n* = 6 master’s level, *n* = 4 non-masters). A total of 30 cases were completed with a master’s-level parent coach and 49 cases completed ABC with a non-master’s-level coach. The study was reviewed and approved by the University’s Institutional Review Board.

### Procedure

Participants were referred from CPS if a child was not placed into foster care. Research staff independently reached out to caregivers to enroll families in the study. Families were eligible if they had a child between 6 and 24 months old and the caregiver had custody of the child. Parent–child dyads were then randomized to receive either ABC or a matched control intervention, Developmental Education for Families (DEF). Dyads randomized to DEF were not included in the sample due to the nature of the research question. For more information about ABC, please see [9].

Post-intervention assessments were completed one month after the completion of ABC and/or at child age of 12 and 24 months. These follow-up research appointments included both a home and an in-lab research visit. There were 79 dyads (master’s = 6, non-master’s = 4) who completed a first post-intervention play assessment (e.g., one month post-intervention, 12 months of age, or 24 months of age), 62 dyads (master’s = 4, non-master’s = 4) completed an assessment with the Strange Situation (either 12 or 24 months of age), and 42 dyads (master’s = 5, non-master’s = 4) completed a 36-month assessment. We considered this to be our analytic sample for evaluating comparative effectiveness of maternal sensitivity, intrusiveness, and positive regard between master’s and non-master’s clinicians.

### Measures

*Parental sensitivity, intrusiveness, and positive regard* were observed pre-and post-intervention in an unstructured free play assessment conducted in participants’ homes with a standardized set of toys. Videos were coded for parental sensitivity, intrusiveness, and positive regard using a coding system adapted from the National Institute of Child Health and Human Development Study of Early Childhood Care. Further detail can be found in Bick and Dozier [12] and Bernard and colleagues [11]. The videos were double coded (ICC = .70), and scores were averaged across double coders for analyses. If multiple assessments of parenting behaviors were available (e.g., post-intervention, 12 months, and 24 months), the first post-intervention session was used, and child age at the time of the first session was used as a covariate to control for any developmental differences.

*Attachment organization* was assessed using the gold-standard paradigm, the Strange Situation. The paradigm is approximately 20 minutes during which the parent separates from and reunifies with the child two separate times. Attachment behaviors were coded during the reunifications using established criteria by two expert coders with good interrater reliability (*k* = .76). Children were classified as organized or disorganized. Disorganized classification was met if the child showed behaviors such as misdirected attachment cues (e.g., approaching the stranger when distressed), stereotypies or abnormal or rigid posture, freezing or stilling, or fear directed toward the parent. Bernard and colleagues discuss this in further detail [20]. Attachment was assessed at the 12-or 24-month assessment.

*Inhibitory control* was measured when children were 36-months old. The children participated in a lab task where children were told not to touch enticing toys on the shelf next to them. The task was recorded from behind a two-way mirror and were subsequently coded for latency to touch the toys, in seconds. Twenty percent of the videos were double coded and showed good interrater reliability (*κ* =.85). See Lind and colleagues^21^ for more detail. Inhibitory control was assessed at the 36-month assessment.

### Analytic plan

Student‘s t-tests and Chi-square or Fisher’s Exact tests were used where appropriate to evaluate group differences between master’s-level and non-master’s-level parent coaches. Data were analyzed using the statistical software R, version 4.2.2 (R Core Team, 2022). Due to the nested nature of the sample, ABC cases nested within parent coaches, linear or logistic mixed models were conducted in R using the *lme4* package [13]. Effect sizes were calculated with the “effectsizes” package in R [14]. Partial eta-squared (*ηp* ^2^) were used as the effect size for continuous outcomes, and odds ratios were used as the effect size for binary outcomes. Non-inferiority was assessed by setting a delta threshold of half a standard deviation for continuous outcomes and 20% for binary outcomes. Non-inferiority was met if the delta was greater than the lower end confidence interval. Unless otherwise indicated, analyses used *α* = 0.05 to evaluate significance.

## Results

### Preliminary analyses

Parents who received ABC from non-master’s-level parent coaches did not significantly differ from parents with master’s-level coaches in pre-intervention sensitivity, intrusiveness, and positive regard *(p* values > .05). Additionally, the number of intervention sessions completed or number of cases that successfully completed the intervention did not differ across provider type (*p* values > .05). Child characteristics did not differ between non-master’s-level and master’s-level coaches (Table 1).

**Table 1.** Child demographics broken down by master’s-level and non-master’s-level parent coaches

	Master’s level (*n* = 31 children; 6 parent coaches)	Non-masters level (*n* = 48 children; 4 parent coaches)	*p*-Value
**Average child age at start of intervention, in months (SD)**	11.86 (6.52)	11.00 (7.05)	0.929
**Child sex – *n* (%)**			0.207
Male	20 (64.52%)	24 (50.00%)	
Female	11 (35.48%)	24 (50.00%)	
**Child Race – *n* (%)**			0.677
Black/African American	19 (61.29%)	34 (70.83%)	
White	3 (9.68%)	6 (12.50%)	
Biracial	9 (29.03%)	7 (14.58%)	
Other	0 (0.00%)	0 (0.00%)	
Unknown/Refused to Answer	0 (0.00%)	1 (2.08%)	
**Child ethnicity - *n* (%)**			0.805
Non-Hispanic or Latino	27 (87.10%)	40 (83.33%)	
Hispanic or Latino	4 (12.90%)	7 (14.58%)	
Unknown/refused to answer	0 (0.00%)	1 (2.08%)	
**Average caregiver age (SD)**	28.30 (7.55)	28.05 (9.51)	0.902
**Income**			0.598
Less than $10,000	17 (54.84%)	26 (54.17%)	
$10,000–$19,999	6 (19.35%)	5 (10.42%)	
$20,000–$29,999	5 (16.13%)	5 (10.42%)	
$30,000–$39,999	1 (3.23%)	3 (6.25%)	
$40,000–$59,999	1 (3.23%)	1 (2.08%)	
$60,000–$99,999	0 (0.00%)	0 (0.00%)	
Not Reported/Unknown	1 (3.23%)	8 (16.67%)	
**Parental Education-*n* (%)**			0.808
Some high school	23 (74.19%)	30 (62.50%)	
High school diploma or GED	8 (25.81%)	10 (20.83%)	
Some college	0 (0.00%)	3 (6.25%)	
4-year college degree	0 (0.00%)	1 (2.08%)	
Post-graduate degree	0 (0.00%)	1 (2.08%)	
Unknown/refused to answer	0 (0.00%)	3 (6.25%)	

Table 1 Child Demographics broken down by master’s level and non-master’s-level parent coaches. Continuous outcomes were evaluated with multi-level models with children nested within parent coaches and categorical outcomes were evaluated with clustered chi-squared or logistic multi-level models. Due to reduced cell counts for child race, it was collapsed into marginalized and non-marginalized to run a logistic multi-level model. Income was collapsed into 3 categories (less than $10,000; $10,000–$19,999; greater than $20,000). Parental education was collapsed into some high school and high diploma/GED and beyond.

### Primary analyses

The first post-intervention visit completed varied by age, so child age at the first post-intervention visit was retained as a covariate in models. On average, maternal sensitivity and maternal positive regard increased, and intrusiveness decreased. At the post-intervention follow-up visits, there were no significant differences between parents who received ABC from master’s-level and non-masters-level parent coaches in parental sensitivity (*B* = -0.02, SE = 0.25, *p* = .933), intrusiveness (*B* = 0.27, SE = 0.32, *p* = .410), or positive regard (*B* = 0.07, SE = 0.30, *p* = .828). After adding pre-intervention scores and child age at first post-intervention visit as covariates in the models to assess change from pre-to post-intervention, there were no significant differences between parents who were trained by master’s-level and non-masters-level parent coaches in sensitivity (*p* = .785), intrusiveness (*p* = .540), and positive regard (*p* = .881). It should be noted that the model decreased in sample size between the unadjusted (*N* = 71) and adjusted models (*n* = 53) due to missingness at the predictor level. The effect sizes, calculated with estimated partial eta-squared, were small (*ηp* ^2^ = 0.001 0.007, <0.001, respectively). Non-inferiority was met for sensitivity (Δ = 0.44, 95% CI [−0.42, 0.56]) and intrusiveness (Δ = 0.56, 95% CI [−0.42, 0.93]). Non-inferiority was not met for positive regard (Δ = 0.56, 95% CI [−0.61, 0.72]).

Child age at the time of the Strange Situation and at the time of the inhibitory control task did not vary by the predictor (i.e., master’s level versus non-master’s level) or the outcome (attachment organization or latency to touch the toys), so child age was not retained as a covariate in those models. We did not have baseline measures of attachment (or inhibitory control, described below) so we could not measure whether these changed from the pre-intervention assessment. For attachment organization, master’s and non-master’s-level parent coaches did not significantly differ (B = -0.67, SE = 0.58, *p* = .244), but the master’s-level parent coaches had lower odds of the children being classified as disorganized compared to the non-master’s-level parent coaches (OR = .51). Non-inferiority was not met (Δ = 20% difference).

For inhibitory control, there was a non-significant difference between master’s and non-masters on latency to touch the toys in seconds (*B* = −1.67, SE = 29.72, *p* = .955) with a very small effect size (*ηp* ^2^ < .001). However, non-inferiority of non-masters compared to masters was not met (Δ = 47.26, 95% CI [−59.83, 56.50]). Master’s-level parent coaches did not significantly differ from non-master’s parent coaches on total duration of touching the toys (*B* = −0.30, SE = 0.40, *p* = .455), with a small effect size (*ηp* ^2^ = .01). Non-inferiority was not met (Δ = 0.70, 95% CI [−1.10, 0.49]). Table 2 displays model parameters.

**Table 2.** Model estimates and parameters

	Sensitivity		Intrusiveness		Positive regard		Attachment organization		Inhibitory control	
	Beta (SE)	*p*-value	Beta (SE)	*p*-value	Beta (SE)	*p*-value	Beta (SE)	*p*-value	Beta (SE)	*p*-value
Intercept	0.91 (.44)	0.042	5.12 (.56)	<0.001	2.31	<0.001	−0.43	0.198	85.30	<0.001
Masters	0.07 (.25)	0.785	0.20	0.539	0.05	0.881	−0.67	0.244	−1.67	0.955
Pre-intervention	0.48 (.14)	<0.001	−0.51	0.005	−0.02	0.004	⎯	⎯	⎯	⎯
Child age	0.03 (.02)	0.072	−0.07	0.002	−0.02	0.395	⎯	⎯	⎯	⎯
*n* children	53		53		53		62		42	
*n* parent coaches	8 (master’s = 4, non-master’s = 4)		8 (master’s = 4, non-master’s = 4)		8 (master’s = 4, non-master’s = 4)		8 (master’s = 4, non-master’s = 4)		9 (master’s = 5, non-master’s = 4)	

Table 2 Model estimates and parameters. Child age at the time of the strange situation or at the time of the inhibitory control task did not vary by the predictor (i.e., master’s level versus non-master’s level) or the outcome (attachment organization or latency to touch the toys), so it was not retained as a covariate in those models.

## Discussion

The goal of this comparative effectiveness study was to test whether peer supports were non-inferior to master’s-level parent coaches at delivering the Attachment and Biobehavioral Catchup intervention. We found that peer supports or non-master’s-level parent coaches were non-inferior to master’s-level parent coaches for parental sensitivity and intrusiveness. Although our hypotheses were not supported for positive regard, attachment organization, and inhibitory control, master’s-level parent coaches did not significantly predict more improved parental or child outcomes in these areas. Therefore, questions remain regarding whether parent coach training level differs in effectiveness for these attachment and child behavior outcomes.

Consistent with study hypotheses, education level of the interventionist was not significantly associated with improvements in parental sensitivity, intrusiveness, or positive regard. In other words, parents became more sensitive, showed more positive regard/warmth, and less intrusive parenting, regardless of whether their parent coach had a master’s degree or not. This finding is important because it shows that improvements in parenting behaviors may be achieved without requiring highly specialized or advanced degrees from interventionists. This finding is consistent with prior work showing that, assuming they receive high-quality parenting intervention training, lay health workers, including peer specialists, are effective at delivering parenting interventions [15].

There were no significant differences in the rates of secure, insecure, and avoidant attachment organization for master’s and non-master’s-level clinicians. However, master’s-level parent coaches were less likely to have toddlers classified as disorganized compared to non-master’s-level clinicians, and non-inferiority between provider types was not met. Clinician education level may play a more important role for complex, dyadic processes. Master’s-level clinicians may have been trained to assess for disorganized attachment behaviors throughout the therapeutic process and could have coached parents on reducing their threatening or threatened behavior, and enhanced their sensitivity, in order to reduce the likelihood that a disorganized attachment develops. On the other hand, given that attachment quality was not assessed at baseline, these outcomes could also be due to spurious differences between provider groups since families were not randomized at the provider level.

Parent coach education level also did not affect the degree to which children exhibited inhibitory control as preschoolers. However, non-inferiority was not met for this child behavior outcome. This finding supports the working hypothesis that, for higher-ordered skills, such as executive functioning, additional provider education level and training may be important for achieving treatment gains. Parent coaches with a master’s degree may have been particularly skilled at supporting parents to support toddler regulation, which could have led to higher, but non-significant, gains in inhibitory control for this group. Future well-powered clinical trials randomized at the provider level will help to more rigorously evaluate this hypothesis.

### Implications for clinical and translational research

The clinical implications of these findings are noteworthy. Many SUD treatment programs struggle to meet the demands of their communities, and many parents in treatment for substance use disorder are challenged to find high-quality, evidence-based treatments [16,17]. This study provides initial evidence that peer support specialists can be trained to deliver ABC in a manner that is comparable to master’s-level clinicians for key mechanisms that have been shown to mediate the effects of ABC on child outcomes [18]. Specifically, equivalent improvements in parental sensitivity were observed across ABC provider type. This information could expand parenting intervention access for parents with substance use disorder and to increase the capacity to provide care at community treatment centers.

These findings also have important translational implications for how “laboratory-grown” interventions can be implemented in the community. Input from our partners with lived experience who were consulted in the development of this re-analysis made it clear that parenting interventions for parents with substance use disorders may be received more comfortably and with less stigma if delivered by a parent coach with a history of substance use. Indeed, this is precisely why many community mental health centers rely on individuals with lived experience to support families in distress [19,20]. While larger, implementation-science-informed trials are needed to fully test this hypothesis, this study provides initial evidence that these larger trials could lead to significant improvements in parenting and child behavior.

### Limitations and future directions

These data were obtained from existing ABC trials which were not originally designed to compare the effectiveness of ABC as delivered by different provider types. Thus, the evaluation of parent coach type involves secondary data analysis, and other design considerations would have been made if the goal was a comparative effectiveness trial. For instance, it may be that the more challenging clinical cases were assigned to master’s-level parent coaches. However, there were no significant differences in parental sensitivity at baseline between master’s-level and non-mater’s-level clinicians, mitigating this concern in the current sample. Nevertheless, future trials should randomly assign cases to master’s-level clinicians and peer support specialists.

This study provides initial evidence to support the hypothesis that peer support parent coaches are as effective as master’s-level clinicians at delivering the Attachment and Biobehavioral Catchup intervention. To increase capacity for parenting supports, treatment providers could consider training peer support specialists to deliver ABC, as they should be just as effective as master’s-level clinicians at enhancing maternal sensitivity and promoting child behavior regulation.
